# Supplementary figures and images for: A mutant form of Dmc1 that bypasses the requirement for accessory protein Mei5-Sae3 reveals independent activities of Mei5-Sae3 and Rad51 in Dmc1 filament stability
Source: PLoS Genet. 2019 Dec 2;15(12):e1008217. doi: 10.1371/journal.pgen.1008217 (PMC6907854; doi:10.1371/journal.pgen.1008217)

|                   | Dmc1 6 hr                                                                         | Control                                                                           | Expression relative<br>to wild-type |
|-------------------|-----------------------------------------------------------------------------------|-----------------------------------------------------------------------------------|-------------------------------------|
| wild-type         | 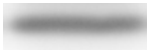 | 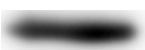 | N.A.                                |
| <i>dmc1-E157D</i> | 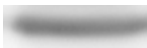 | 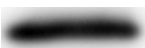 | 0.95                                |

Supplement: S1 Fig — Left column, W. blot against Dmc1 for 5μL sample prepared from meiotic yeast cultures at 6 hours as described in Methods Section for each strain. Control column is 20 ng purified Dmc1 protein that was run in parallel with sample and used to quantitate blots. Sample concentration is estimated concentration in comparison to 20 ng purified Dmc1 protein. Similar results were obtained from an independent meiotic time course. Strains used in this experiment in the order in which they appear in figure, top to bottom: DKB3698, DKB6342. (PDF) [file pgen.1008217.s001.pdf]

A

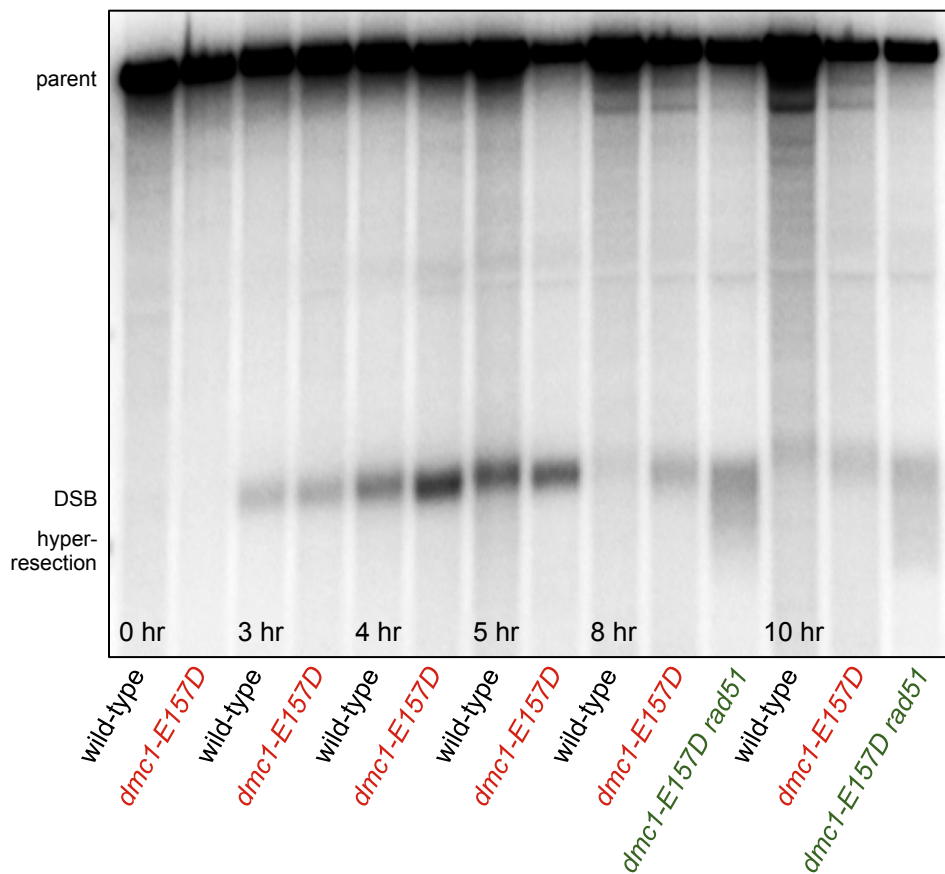

B

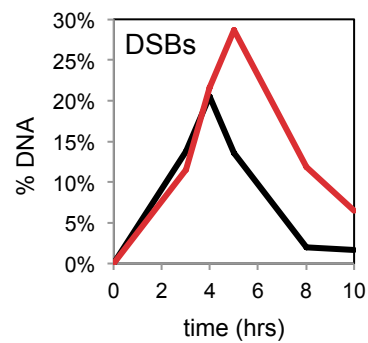

Supplement: S2 Fig — (a) Southern blot analysis at the HIS4::LEU2 hotspot following digestion of genomic DNA from meiotic time course experiments with PstI. Time points and strains are indicated beneath each lane. Note that dmc1-E157D rad51 is shown at late time points as a reference for hyper-resection. (b) Quantitation of 1D gels shown in (a); black–wild-type, red–dmc1-E157D. Strains used in experiments in the order in which they appear in figure, left to right: DKB3698, DKB6342, DKB6393. (PDF) [file pgen.1008217.s002.pdf]

A

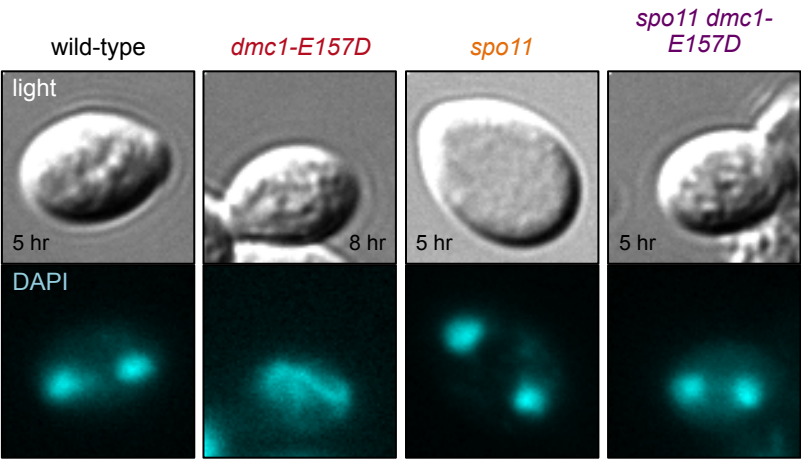

B

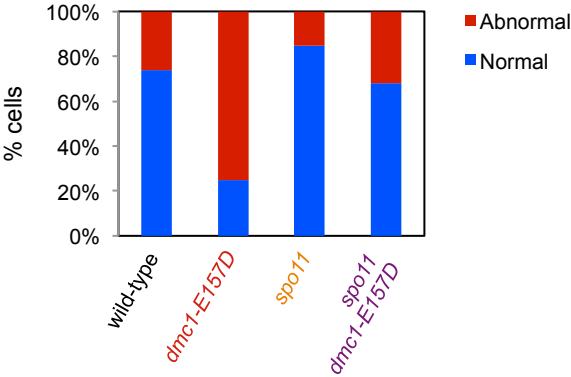

C

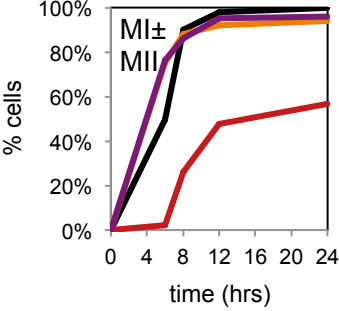

Supplement: S3 Fig — (a) Representative images showing MI segregation in the strains indicated. (b) Quantitation of MI division defects observed in the strains indicated. A “normal” division is defined as having two equally-sized and well-defined DAPI staining bodies, whereas an “abnormal” division is defined as having unequal DAPI bodies or DNA connecting the two DAPI bodies. (c) Meiotic progression data for strains indicated. For each time point, ≥50 cells were scored. Strains used in this experiment in the order in which they appear in figure, top to bottom: DKB3698, DKB2123, DKB6342, DKB6419. (PDF) [file pgen.1008217.s003.pdf]

A

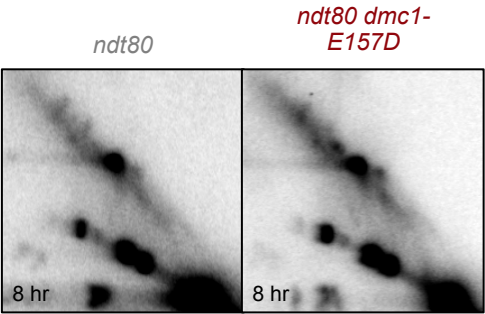

B

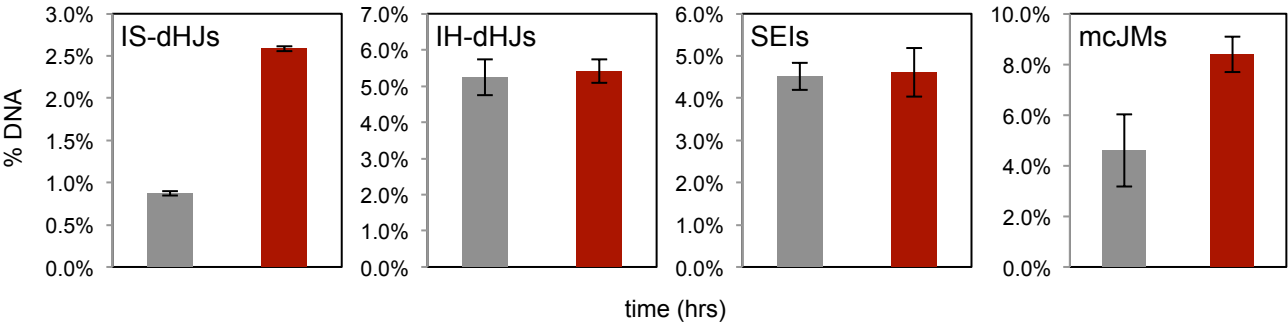

Supplement: S4 Fig — (a) Southern analysis at the HIS4::LEU2 hotspot at 8 hours following 2D gel electrophoresis. (b) 2D gel quantitation; dark gray–ndt80, dark red–ndt80 dmc1-E157D. Quantitation for each strain represents an average of two independently cultured diploids. Strains used in this experiment in the order in which they appear in figure, left to right: DKB3689, DKB3428, DKB6676, DKB6682. (PDF) [file pgen.1008217.s004.pdf]

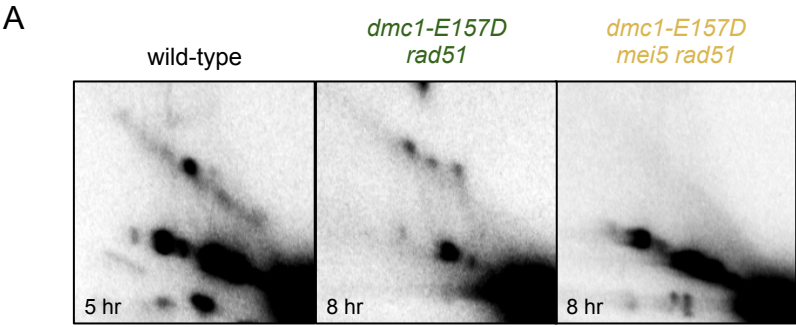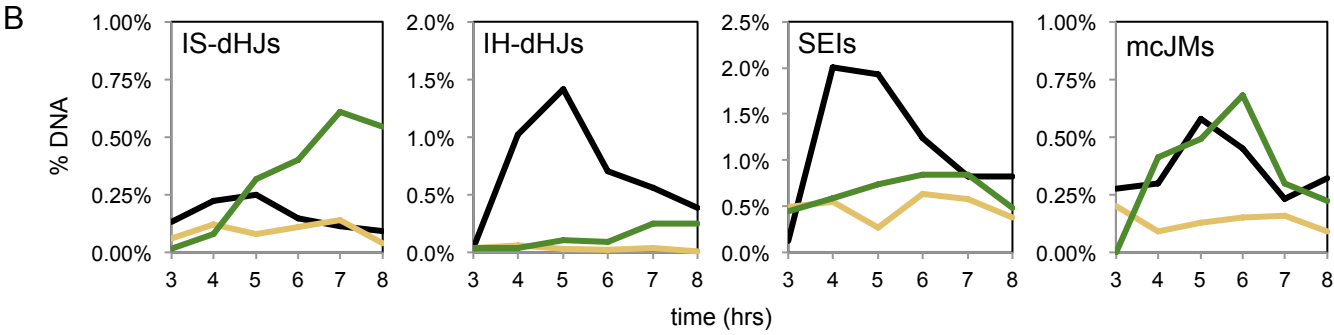

Supplement: S5 Fig — For dmc1-E157D mei5 rad51, two independently derived diploids were used. (a) Southern analysis at the HIS4::LEU2 hotspot from meiotic time course experiments following 2D gel electrophoresis. Time point for representative image is shown in the bottom left corner. (b) 2D gel quantitation; black–wild-type, dark green–dmc1-E157D rad51, yellow–dmc1-E157D mei5 rad51. Strains used in this experiment in the order in which they appear in figure, left to right: DKB3698, DKB6393, DKB6413. (PDF) [file pgen.1008217.s005.pdf]

A

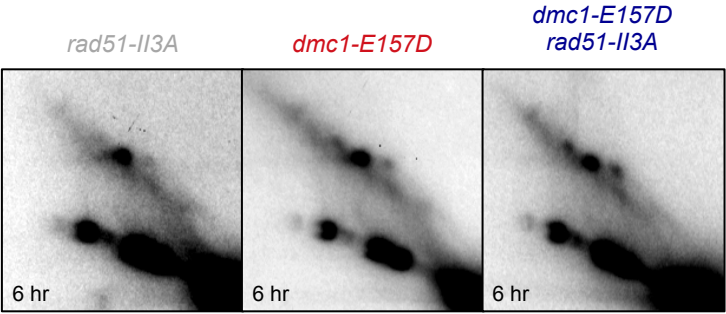

B

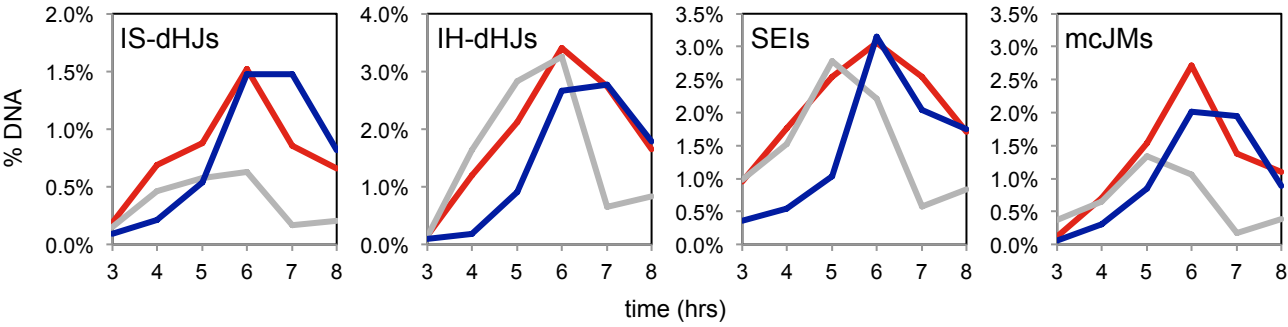

Supplement: S6 Fig — (a) Southern analysis at the HIS4::LEU2 hotspot from meiotic time course experiments following 2D gel electrophoresis. Time point for representative image is shown in the bottom left corner. (b) 2D gel quantitation; gray–rad51-II3A, red–dmc1-E157D, dark blue–dmc1-E157D rad51-II3A. Strains used in this experiment in the order in which they appear in figure, right to left: DKB3689, DKB6342, DKB6400. (PDF) [file pgen.1008217.s006.pdf]

A

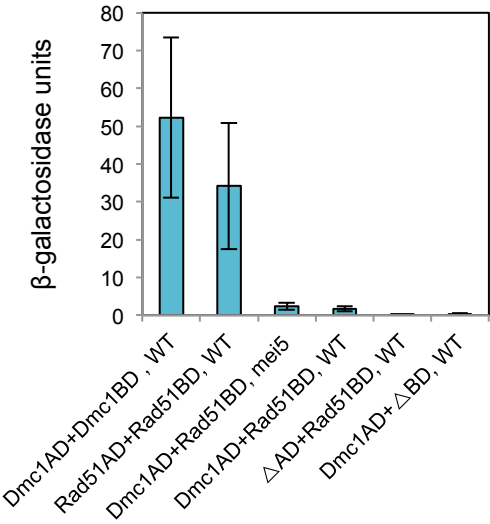

B

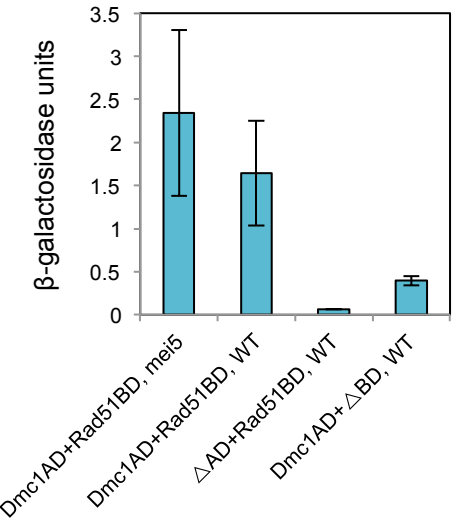

Supplement: S7 Fig — (a) All interactions examined are plotted. (b) Subset of the same data shown in (a) to facilitate comparison of measurements of the Rad51-Dmc1 interaction with empty vector controls. The difference between Dmc1AD+Rad51BD mei5 and Dmc1AD+Rad51BD WT (wild-type) is not statistically significant (p = 0.5 using a Wilcoxon signed-rank test). ΔBD and ΔAD represent the empty vectors. Strains used in this experiment: DKB6501, DKB6503, DKB6508, DKB6509, DKB6513, DKB6515. (PDF) [file pgen.1008217.s007.pdf]

A

confocal

deconvolved  
STED

*dmc1-E157D*  
*mei5*

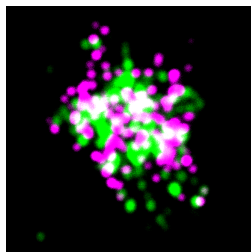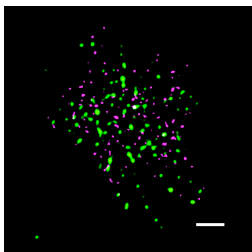

B

RPA

Dmc1

Merge

*spo11 dmc1-*  
*E157D*

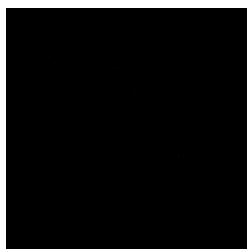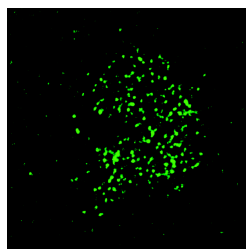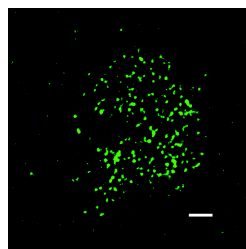

Supplement: S8 Fig — (a) Spread meiotic nuclei prepared from a dmc1-E157D mei5 5 hour sample imaged using confocal and STED microscopy methods. (b) STED imaging of a spo11 dmc1-E157D spread meiotic nuclei at 5 hours. For both, scale bar represents 1 micrometer. Magenta, RPA, green, Dmc1. Strains used in this experiment: DKB6300, DKB6419. (PDF) [file pgen.1008217.s008.pdf]

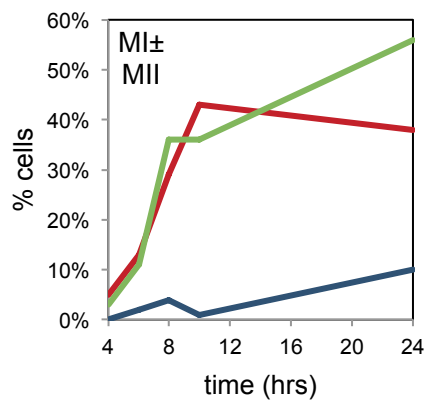

*rdh54*

*dmc1-E157D*

*dmc1-E157D rdh54*

Supplement: S9 Fig — Meiotic progression data for strains indicated. For each time point, ≥100 cells were scored. Strains used in this experiment in the order in which they appear in figure, top to bottom: DKB2526, DKB6342, DKB6583. (PDF) [file pgen.1008217.s009.pdf]

*spo11*

*spo11 rdh54*

*spo11 rdh54 mei5*

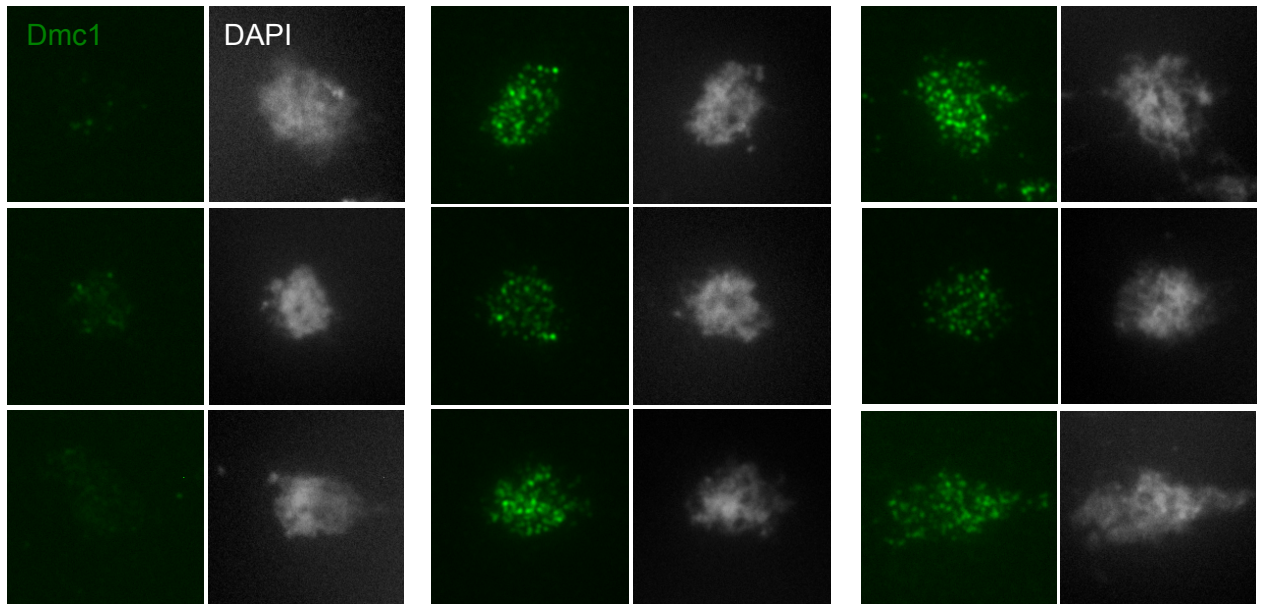

Supplement: S10 Fig — Samples were collected 4 hours after induction of meiosis in liquid medium and immuno-stained for Dmc1 and Hop2. Because Hop2 staining is Spo11 independent and specific for meiotic prophase, random prophase nuclei were selected on the basis of being Hop2 positive and then imaged for Dmc1 staining. 50 nuclei were examined for each sample with 3 representative nuclei shown for each of the three strains examined. Images were generated by wide-field microscopy using the same camera settings for all strains. Strains used in this experiment in the order in which they appear in figure, top to bottom: DKB2524, DKB2523, and DKB6571. (PDF) [file pgen.1008217.s010.pdf]
