# Supplementary material for: A mutant form of Dmc1 that bypasses the requirement for accessory protein Mei5-Sae3 reveals independent activities of Mei5-Sae3 and Rad51 in Dmc1 filament stability
Source: PLoS Genet. 2019 Dec 2;15(12):e1008217. doi: 10.1371/journal.pgen.1008217 (PMC6907854; doi:10.1371/journal.pgen.1008217)
Supplement: S1 Table — (PDF) [file pgen.1008217.s011.pdf]

Name	Strain	Genotype
wild-type	DKB3698	*ho::hisG/", leu2::hisG/", ura3(ΔSma-Pst)/", HIS4::LEU2-(BamHI; +ori)/his4-X::LEU2-(NgoMIV; +ori)-URA3*
dmc1-E157D	DKB6342	*lys2/", ho::hisG/ho::LYS2, leu2::hisG, ura3/", HIS4::LEU2-(BamHI; +ori)/his4-X::LEU2-(NgoMIV; +ori)-URA3, dmc1-E157D-NATMX4/"*
DMC1⁺/dmc1-E157D	DKB6398	*LYS2/lys2 or LYS2, ho::hisG/ho::hisG or ho::LYS2, leu2::hisG/", ura3/", HIS4::LEU2-(BamHI; +ori)/", dmc1-E157D-NATMX4/DMC1⁺*
DMC1⁺/dmc1-E157D	DKB6399	*LYS2/lys2 or LYS2, ho::hisG/ho::hisG or ho::LYS2, leu2::hisG/", ura3/", HIS4::LEU2-(BamHI; +ori)/", dmc1-E157D-NATMX4/DMC1⁺*
mei5	DKB6320	*ho::hisG/", leu2::hisG/", ura3/", HIS4::LEU2-(BamHI; +ori)/his4-X::LEU2-(NgoMIV; +ori)-URA3, mei5::KANMX/"*
ndt80	DKB3428	*ho::hisG/", leu2::hisG/", ura3(ΔPst-Sma)/", HIS4X::LEU2-(BamHI)-ura3/his4::LEU2(NgoMIV; +ori)-URA3, ndt80::KANMX4/"*
rad51	DKB3710	*ho::hisG or LYS2/", ura3/", leu2::hisG/", HIS4::LEU2-(BamHI; +ori)/his4X::LEU2-(NgoMIV; +ori)-URA3, rad51::hisG/"*
rad51-II3A	DKB3689	*ho::hisG/", leu2::hisG/", ura3(ΔSma-Pst)/",HIS4-X::LEU2-(BamHI; +ori)-ura3/his4X::LEU2-(NgoMIV; +or)-URA3, RAD51-R188A, K361A, K371A-KANMX6/"*
rdh54	DKB2526	*ho::LYS2/", lys2/", leu2::hisG/", his4-X::LEU2/", trp1::hisG/", tid1::LEU2/"*
spo11	DKB2123	*ho::LYS2/", lys2/", leu2::hisG/", ura3/", his4-X/his4B, spo11::hisG-URA3-hisG/"*
spo11	DKB2524	*ho::LYS2/", lys2/", leu2::hisG/", trp1::hisG/", ura3/", his4X::LEU2/", spo11::hisG-URA3-hisG/"*
dmc1-E157D mei5	DKB6299	*ho::LYS2/ho::hisG, ura3/", leu2::hisG/", HIS4::LEU2-(BamHI; +ori)/his4-X::LEU2-(NgoMIV; +ori)-URA3, dmc1-E157D-NATMX4/", mei5::KANMX/"*
dmc1-E157D mei5	DKB6300	*ho::LYS2/ho::hisG, ura3/", leu2::hisG/", HIS4::LEU2-(BamHI; +ori)/his4-X::LEU2-(NgoMIV; +ori)-URA3, dmc1-E157D-NATMX4/", mei5::KANMX/"*
DMC1/dmc1-E157D mei5	DKB6406	*ho::hisG, leu2::hisG, ura3, his4-X::LEU2-(NgoMIV; +ori)-URA3/HIS4::LEU2-(BamHI; +ori), mei5::KANMX/", dmc1-E157D-NATMX4/DMC1⁺*
DMC1/dmc1-E157D mei5	DKB6407	*ho::hisG, leu2::hisG, ura3, his4-X::LEU2-(NgoMIV; +ori)-URA3/HIS4::LEU2-(BamHI; +ori), mei5::KANMX/", dmc1-E157D-NATMX4/DMC1⁺*
dmc1-E157D ndt80	DKB6676	*ho::hisG/", lys2/", leu2::hisG/", ura3/", HIS4X::LEU2-(BamHI)-ura3/his4::LEU2(NgoMIV; +ori)-URA3, ndt80::KANMX4/", dmc1-E157D-NATMX4/"*
dmc1-E157D ndt80	DKB6682	*LYS2/lys2 or LYS2, ho::hisG/ho::hisG or ho::LYS2, leu2::hisG/", ura3/", HIS4X::LEU2-(BamHI)-ura3/his4::LEU2(NgoMIV; +ori)-URA3, ndt80::KANMX4/", dmc1-E157D-NATMX4/"*
dmc1-E157D rad51	DKB6393	*lys2* or *LYS2/", ho::hisG* or *ho::LYS2/", ura3/", leu2::hisG/", arg4-nsp/ARG4, his4-X::LEU2-(NgoMIV; +ori)--URA3/HIS4::LEU2-(BamHI; +ori), dmc1-E157D-NATMX4/", rad51::hisG/"*
dmc1-E157D rad51-II3A	DKB6400	*ho::hisG/", leu2::hisG/", ura3/", HIS4::LEU2-(BamHI; +ori)/his4-X::LEU2-(NgoMIV; +ori)-URA, RAD51-R188A, K361A, K371-A-KANMX6/", dmc1-E157D-NATMX4/"*
dmc1-E157D rdh54	DKB6583	*ho::LYS2/", lys2/", leu2::hisG/", his4-X::LEU2/", TRP1/trp1::hisG, tid1::LEU2/", dmc1-E157D-NATMX4/"*
dmc1-E157D sae3	DKB6539	*lys2/", ho::LYS2/", leu2 or LEU2/", ura3/", HIS4::LEU2-(BamHI; +ori)/his4-X::LEU2-(NgoMIV; +ori)-URA3, dmc1-E157D-NATMX4/", sae3::hisG-URA3-hisG/"*
dmc1-E157D sae3	DKB6540	*lys2/", ho::LYS2/", leu2 or LEU2/", ura3/", HIS4::LEU2-(BamHI; +ori)/his4-X::LEU2-(NgoMIV; +ori)-URA3, dmc1-E157D-NATMX4/", sae3::hisG-URA3-hisG/"*
spo11 dmc1-E157D	DKB6419	*ho::hisG* or *ho::LYS2/ho::hisG, LYS2* or *lys2/lys2, leu2::hisG/", ura3/", his4-X::LEU2-(NgoMIV; +ori)-URA3/HIS4::LEU2-(BamHI; +ori), dmc1-E157D-NATMX4/", spo11::hisG-URA3-hisG/"*
spo11 rdh54	DKB2523	*ho::LYS2/", lys2/", ura3/", leu2::hisG/", his4-X::LEU2/", trp1::hisG/", tid1::LEU2/", spo11::hisG-URA3-hisG/"*
dmc1-E157D mei5 rad51	DKB6412	*lys2 or LYS2/", ho::hisG or ho::LYS2/", ura3/", leu2::hisG/", arg4-nsp or ARG4/ARG4, HIS4::LEU2-(BamHI; +ori)/his4-X::LEU2-(NgoMIV; +ori), rad51::hisG/", dmc1-E157D-NATMX4/", mei5::KANMX/"*
dmc1-E157D mei5 rad51	DKB6413	*lys2* or *LYS2/", ho::hisG* or *ho::LYS2/", ura3/", leu2::hisG/", arg4-nsp/ARG4, HIS4::LEU2-(BamHI; +ori)/his4-X::LEU2-(NgoMIV; +ori), rad51::hisG/", dmc1-E157D-NATMX4/", mei5::KANMX/"*
spo11 dmc1-E157D mei5	DKB6425	*ho::hisG or ho::LYS2/", lys2 or LYS2, leu2::hisG/", ura3/", HIS4::LEU2-(BamHI; +ori)/his4-X::LEU2-(NgoMIV; +ori)-URA3, dmc1-E157D-NATMX4/", spo11::hisG-URA3-hisG/", mei5::KANMX/"*

Name	Strain	Genotype
spo11 mei5 rdh54	DKB6571	*ho::LYS2/", lys2/", ura3/", leu2::hisG/", his4-X::LEU2/", trp1::hisG/", tid1::LEU2/", spo11::hisG-URA3-hisG/", mei5::KANMX/"*
two-hybrid strain	DKB6501	*lys2/", ho::LYS2/", URA3/", leu2:hisG/", his4-X/HIS4, trp1::hisG/", arg4-nsp or ARG4/", dmc1::ARG4/", rad51::hisG/", ndt80::KANMX/", LexA(op)-lacZ::URA3/"* +pNRB729 +pNRB271
two-hybrid strain	DKB6503	*lys2/", ho::LYS2/", URA3/", leu2:hisG/", his4-X/HIS4, trp1::hisG/", arg4-nsp or ARG4/", dmc1::ARG4/", rad51::hisG/", ndt80::KANMX/", LexA(op)-lacZ::URA3/"* +pNRB727 +pNRB688
two-hybrid strain	DKB6508	*lys2/", ho::LYS2/", URA3/", leu2:hisG/", HIS4/his4-X, trp1::hisG/", arg4-nsp or ARG4/", dmc1::ARG4/", :mei5:KANMX/", rad51::hisG/", ndt80::KANMX/", LexA(op)-lacZ::URA3/"* +pNRB727 +pNRB271
two-hybrid strain	DKB6509	*lys2/", ho::LYS2/", URA3/", leu2:hisG/", his4-X/HIS4, trp1::hisG/", arg4-nsp or ARG4/", dmc1::ARG4/", rad51::hisG/", ndt80::KANMX/", LexA(op)-lacZ::URA3/"* +pNRB727 +pNRB271
two-hybrid strain	DKB6513	*lys2/", ho::LYS2/", URA3/", leu2:hisG/", his4-X/HIS4, trp1::hisG/", arg4-nsp or ARG4/", dmc1::ARG4/", rad51::hisG/", ndt80::KANMX/", LexA(op)-lacZ::URA3/"* +pNRB728 +pNRB271
two-hybrid strain	DKB6515	*lys2/", ho::LYS2/", URA3/", leu2:hisG/", his4-X/HIS4, trp1::hisG/", arg4-nsp or ARG4/", dmc1::ARG4/", rad51::hisG/", ndt80::KANMX/", LexA(op)-lacZ::URA3/"* +pNRB727 +pNRB267
